# Public thresholds for antidepressant effectiveness and adverse drug reaction rates: a cross-sectional study

**DOI:** 10.1038/s41598-026-62116-y

**Published:** 2026-07-15

**Authors:** Katharina Apel, Jennifer Scheel-Barteit, Matthias Berking, Thomas Kühlein, Maria Sebastião

**Affiliations:** 1 Institute of General Practice, Friedrich-Alexander-University Erlangen- Nuremberg, Erlangen (Bavaria), Germany; 2 Department of Clinical Psychology and Psychotherapy, Friedrich-Alexander-University Erlangen-Nuremberg, Erlangen (Bavaria), Germany

**Keywords:** Depressive disorder, Drug-related side effects and adverse reactions, Shared decision making, Members of the public, Minimum acceptable benefit, Diseases, Health care, Medical research

## Abstract

The effectiveness of antidepressants (ADs) in the treatment of depression is continuously discussed regarding its clinical relevance. The objective of this study was to explore thresholds of members of the public for effectiveness of ADs relative to the frequency of adverse drug reactions (ADRs) deemed acceptable. We conducted a cross-sectional online survey in Germany that included individuals with and without experiences of depression and ADs. Participants were presented a case scenario describing a moderate depressive episode, operationalized with the Montgomery Åsberg Depression Rating Scale (MADRS). Participants were asked to rate the effectiveness of different treatment options, estimate the frequency of 15 ADRs, and indicate the minimal level of treatment effectiveness they would require to accept certain ADRs. The survey was completed by 208 participants (144 female, 64 male), 44 were taking ADs. The effectiveness of ADs was significantly overestimated (*t*(204) = 8.96, *p* < 0.001, *d* = 0.63). For 13 ADRs, participants selected package-insert frequency categories that were significantly lower than the corresponding reference categories. For example, to accept the potential ADR of seizures, participants required a symptom improvement of *M* = 23.51 (*SD* = 8.82) points on the MADRS. We propose that patient-physician communication about risks and benefits of AD treatment should explicitly address individual expectations.

**Supplementary Information:**

The online version contains supplementary material available at 10.1038/s41598-026-62116-y.

## Introduction

Depression is one of the most common mental illnesses worldwide^1^. In Germany, the prevalence is estimated between 8.1% and 10.1%^2,3^. For the treatment of a moderate depressive episode, the German National Clinical Practice Guideline for Unipolar Depression (NVL-UD) recommends combined psychotherapy and pharmacological therapy^4^. This recommendation has been subject to sharp critique, as the effectiveness of antidepressants (ADs), though widely utilized in the pharmacological treatment of depressive disorders, remains a matter of considerable debate^5–12^. In particular, the clinical relevance of AD medication is challenged^7,8,12–14^ as ADs show, on average, only little benefits over placebo in clinical trials. The serotonin hypothesis of depression has been questioned and is not strongly supported by the available evidence from multiple lines of research^15^. Despite these controversies and the fact that the use of ADs can be associated with a number of adverse drug reactions (ADRs), such as sexual dysfunction, nausea, headache, dry mouth, sleep disturbance, diarrhea, dizziness, and constipation^16–18^, the volume of AD prescriptions increased by over 40% between 2009 and 2018 in Germany^10^.

In general, the benefits of pharmacological interventions are often overestimated by both physicians and patients^19–21^. Thus, determining the so-called Minimal Important Difference (MID) is essential for evaluating the clinical relevance of ADs^7,22,23^. The concept of MID was introduced into evidence-based medicine, and describes the smallest difference in clinical symptoms required from a patient’s point of view, for the patient to be willing to actively alter their health management, provided there are no ADRs^24^. AD treatment should lead to a symptom reduction of at least 7–9 points on the Montgomery Åsberg Depression Rating Scale (MADRS) to be considered clinically relevant^13,21^. However, the literature indicates that AD treatment typically only results in a reduction of about 3 points on the MADRS^25^. Importantly, previous MID estimates have generally not taken ADRs into account and have largely been based on clinicians’ judgments rather than patients’ or the public’s perspectives. Little is known about how members of the public perceive the effectiveness of ADs or the frequency and acceptability of associated ADRs. Addressing these gaps may contribute to a more comprehensive evaluation of the perceived benefits and harms of AD treatment. As noted by Rossi et al., the MID represents “a low bar”^26^. Patients do not only seek minimal improvement but rather aim to reach a symptom state they perceive as clinically acceptable, which may depend on the presence and severity of adverse drug reactions (ADRs). To further incorporate the patient perspective, we therefore also considered potential ADRs in the present study. This approach reflects the concept of the minimum acceptable benefit (MAB), defined as the smallest improvement in treatment benefit that patients require in order to accept an ADR. Due to the high current and lifetime prevalence of depressive disorders^2,3,27^, it appears highly relevant to also include the perspective of currently non-affected individuals.

In this study, we aimed to explore: (1) How members of the public estimate the effectiveness of ADs, (2) perceptions of ADRs, (3) the level of symptom improvement they demand from an AD to accept certain ADRs (MAB), and (4) which ADRs members of the public would not accept under any circumstances, regardless of AD effectiveness.

## Methods

### Design, recruitment, and data collection

#### Design

We conducted a cross-sectional online survey in Germany using RedCap software^28^, following the CHERRIES checklist^29^(**Supplementary Table 1**). The ethics committee of Friedrich-Alexander-University Erlangen-Nuremberg approved the study in January 2024 (file number: 23-491-B). The survey was developed by the project team at the Institute of General Practice. Pre-testing for design and content evaluation was done with two members of the public using the *“thinking aloud”* method^30^.

#### Recruitment

We recruited participants via different channels such as placing flyers and displaying posters in waiting rooms (psychotherapy practices: *n* = 16, university psychotherapy outpatient clinics: *n* = 5, general practices: *n* = 5), placing flyers and displaying posters at the Wabe e.V (Association for the Reintegration of People with Mental Illness in Erlangen), sharing the link in depression support groups on social media and the website of the Institute for General Practice, sharing the link with the Citizens’ Advisory Board of the Institute for General Practice (*n* = 15). Participants were asked to further spread the link (snowball sampling). Members of the public with and without depression/AD-use were eligible to participate.

#### Data collection

Data were collected anonymously from March 20th to May 1st 2024. Participants accessed the online survey by scanning a QR code on study flyers/posters or clicking the link. Participation was entirely voluntary, with no incentives offered. The only participation requirement was being of legal age. Participants provided informed consent prior to starting the survey by clicking a mandatory check box. The questionnaire comprised seven sections, and participants could change their answers by using a back button (**Supplementary Table 2**). Depending on the filter settings, participants had to complete a maximum of 66 items, which took 10–15 min. The questionnaire was only available in German and could not be completed in any other language. The study was performed in accordance with the Declaration of Helsinki. Due to the open survey design, view and participation rates could not be calculated.

### Measures

#### Sociodemographic characteristics and depression-related information

Data on age, gender, education, current or past depression, and history of AD use were collected. Only participants *with* prior depression and/or AD experience were presented additional questions regarding diagnosis and medication.

#### Effectiveness of treatment options

A case scenario described a moderate depressive episode according to the ICD-10 criteria^31^. Participants were asked to immerse themselves in the scenario and to disregard any symptoms they might themselves be currently experiencing:

*"You have been suffering from a reduced drive for 6 weeks. Activities that you normally enjoy no longer give you any pleasure. You also have difficulty concentrating on things. You have problems falling asleep in the evening. You also wake up several times during the night. You are further troubled by unfounded self-reproaches. You have lost your appetite, so you have already lost three kilograms in the last few weeks".*

**Fig. 1:**
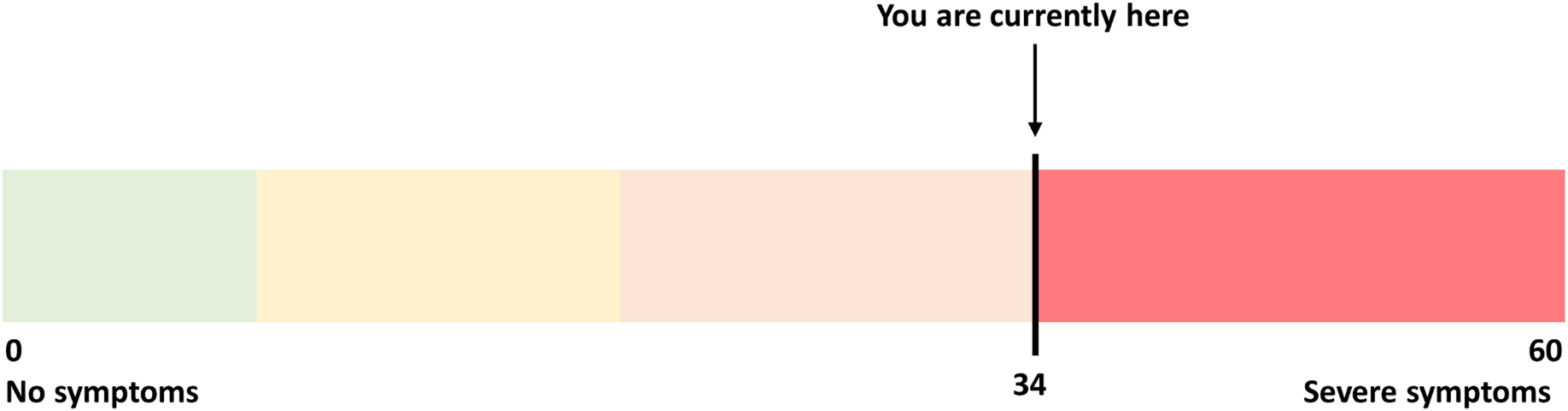
Graphic illustration of the MADRS.

The MADRS was explained and presented graphically (Fig. 1) with the following instruction:

*“Based on your symptoms*,* you are currently at 34 points on the depression scale*,* at the threshold of severe distress. The scale ranges from 0 = no symptoms to 60 = severe symptoms.”*

The slider was color-coded according to the severity categories of the MADRS (0–6: no depression, 7–19: mild depression, 20–34: moderate depression, and 35–60: severe depression).

Participants were asked to use a slider to indicate where they believed they would be after six months of psychotherapy, pharmacological treatment with an AD, combination therapy (psychotherapy and pharmacological treatment), or without any treatment. The effectiveness estimation for *each* treatment form was operationalized by calculating the difference between the participants’ baseline score on the scale (34 points) and their expectations after six months for each treatment form, respectively.

#### Frequency of ADRs

We set the number of ADRs at 15 to keep the survey manageable while covering a wide range of ADRs associated with the AD Citalopram (at 20 mg dosage), ideally affecting different systems (e.g. central nervous system, digestion, psyche). We excluded ADRs that may be difficult to understand (e.g. lethargy). As we assumed that ADRs tend to be overestimated, we only selected ADRs that were very frequent, frequent or occasional. We used the classification system from package inserts, as it is familiar to patients and mandatory for all prescribed medications in Germany. For the final set of ADRs included in this study, see **Supplementary Table 3**. The ADRs were presented in randomized order. Participants were asked to estimate the frequency of occurrence on a 5-point scale (1 = very rare, 2 = rare, 3 = occasionally, 4 = frequent, 5 = very frequent).

To determine the deviation between the estimated and actual frequency according to the prescription information, a difference value was calculated for each ADR. To assess the accuracy of participants’ estimations regarding the frequency of ADRs across all ADRs, the mean of these difference values was calculated. All ADRs were presented in randomized order on a single page.

#### Minimum Acceptable Benefit (MAB)

The MAB required to take an AD was assessed by graphically presenting the MADRS and providing the following instruction:

*“Please recall the previously described scenario. Due to your symptoms (reduced drive*,* loss of pleasure*,* sleep disturbances*,* unfounded self-reproaches*,* loss of appetite)*,* you are at 34 points on the depression scale. Your psychiatrist prescribes an AD*,* which you should take daily. How much would your symptoms need to improve for you to tolerate the associated side effect? Your starting point is 34 points. Please use the slider to indicate the new score to which your symptoms should improve. To move the slider*,* click on it first. If you would not tolerate a side effect under any circumstances*,* please check the corresponding box and do not adjust the slider.”*

For each of the 15 presented ADRs, participants had to use the slider (range: 0–60) to indicate the *lower* score to which their symptoms would need to improve in order to tolerate the ADR, or select that they would not tolerate an ADR at all. Participants who did not accept an ADR were excluded from the MAB analysis for that specific ADR. Again, ADRs were presented in randomized order.

The operationalization of the MAB for taking an AD was achieved by calculating the difference between the baseline score of participants on the MADRS (34 points) and the adjusted score at which they would be willing to tolerate each ADR. Negative values, achieved when respondents increased their score on the slider, were excluded, as they were illogical in the context of the question (**Supplementary Table 4**).

### Statistical analysis

We conducted an explorative analysis. Only questionnaires that were completed through to the end were included in the analyses. Since not all items were mandatory, missing values may occur for individual questions. Descriptive data are provided for all variables (mean and standard deviation). To test if participants significantly overestimate the effectiveness of ADs, a one-sample t-test was conducted. The test value was set at 2.99 based on a review which included a total of 28 RCTs using the MADRS^25^. One-sample t-tests were performed for each individual ADR to compare participants’ estimated frequencies with the actual occurrence rates.

Group comparisons across sex, education level, and with/without experience with depression/ADs were computed with Mann-Whitney-U-Tests for non-normally distributed data. Norman’s review showed that parametric methods, such as calculating difference values, can deliver robust and valid results even with ordinal scaled data^32^.

The software IBM^®^ SPSS^®^ Statistics (Version 29.0.1.0) was used for statistical processing and data analysis. A significance level of = 0.05 was assumed for all calculations. Due to the increased likelihood of Type II errors, we did not adjust for multiple testing (e.g., Bonferroni correction)^33^.

## Results

### Sample characteristics

A total of *N* = 362 participants accessed the survey information and *N* = 333 started to complete the survey. Some participants (*n* = 93) dropped out before responding to the section regarding effectiveness. The final sample consisted of *N* = 208 participants (completion rate: 62%; female: 144, male: 64). This includes *n* = 44 individuals who were concurrently taking or had previously taken ADs (Group 1), and *n* = 164 individuals who were not concurrently taking or had never taken ADs (Group 2) (Table 1).

**Table 1:** Sociodemographic characteristics of the total sample and differentiation by Groups 1 and 2.

Variable	Total Sample (*N* = 208)	Group 1^a^ *(n* = 44)	Group 2^b^ *(n* = 164)
Gender (n, %)			
Female	144 (70)	32 (73)	112 (68)
Male	64 (30)	12 (27)	52 (32)
Age (n, %)			
18–24 years	41 (20)	12 (27)	29 (18)
25–39 years	75 (36)	14 (32)	61 (37)
40–54 years	50 (24)	12 (27)	38 (23)
55–69 years	35 (17)	6 (14)	29 (18)
$\:\ge\:$ 70 years	7 (3)	0 (0)	7 (4)
Highest level of education (n, %)			
Lower secondary school diploma	8 (4)	6 (15)	2 (1)
Secondary school certificate	15 (7)	4 (9)	11 (7)
Completed apprenticeship	21 (10)	3 (7)	18 (11)
High school diploma	56 (27)	15 (34)	41 (25)
University degree	107 (51)	16 (36)	91 (55)
Not specified	1 (1)	0 (0)	1 (1)

^a^Individuals who are currently taking or have previously taken ADs.

^b^Individuals who have not currently or previously taken ADs.

### Effectiveness of antidepressants

Participants rated the combination therapy treatment approach as the most effective (*M* = 17.34, *SD* = 10.19) (Table 2). Participants assumed that moderate depression would improve on average by *M* = 9.92 (*SD* = 11.05) points on the MADRS with ADs. The participants estimated that the symptoms of an untreated depressive episode would worsen on average by *M* = -8.76 (*SD* = 11.14) on the MADRS.

**Table 2:** Descriptive statistics of participants’ effectiveness ratings for different treatments for moderate depression.

Treatment	M	SD	Minimum	Maximum
Psychotherapy	13.08	8.75	-19.00	34.00
Medication therapy	9.92	11.05	-26.00	34.00
Combined therapy	17.34	10.19	-26.00	34.00
No treatment	-8.76	11.14	-26.00	34.00

M = mean, SD = standard deviation.

Negative numbers imply an aggravation of depressive symptoms (maximal aggravation = 26 points on the MADRS (from 34 to 60 points)), positive numbers imply an improvement of depressive symptoms (maximal improvement = 34 points on the MADRS (from 34 to 0 points)).

The t-test revealed a statistically significant difference between the perceived effectiveness of pharmacological therapy and the effectiveness of ADs (test value 2.99), *t*(204) = 8.96, *p* < 0.001, *d* = 0.63. According to Cohen’s conventions^34^, this corresponds to a medium effect size, indicating that the participants significantly overestimated the effectiveness of ADs.

### Estimation of the frequency of ADR occurrence

The probability of the occurrence of the ADR reduced libido was estimated as the highest (*M* = 3.57, *SD* = 1.04), while participants assessed the risk of the ADR fainting as the lowest probability (*M* = 1.74, *SD* = 0.89) (Table 3).

**Table 3:** Descriptive statistics of ADR frequency estimates when undergoing AD treatment.

Variable	M	SD	Minimum	Maximum
Reduced libido	3.57	1.04	1	5
Weight gain	3.55	1.10	1	5
Headache	3.24	1.00	1	5
Palpitations	3.18	1.00	1	5
Sleep disorders	3.07	1.09	1	5
Nausea	3.01	1.03	1	5
Dizziness	2.99	0.97	1	5
Loss of appetite	2.85	1.09	1	5
Mania	2.78	1.13	1	5
Visual disturbances	2.24	1.00	1	5
Hair loss	2.24	1.04	1	5
Hallucinations	2.19	1.12	1	5
Seizures	1.91	0.90	1	5
Fever	1.75	0.85	1	5
Fainting	1.74	0.89	1	5

M = mean, SD = standard deviation.

Rating scale: 1 = very rare, 2 = rare, 3 = occasionally, 4 = frequent, 5 = very frequent.

Table 4 shows the descriptive statistics of the deviations between the estimated and actual frequency of the individual ADRs when undergoing AD treatment.

**Table 4:** Deviations between the estimated and actual frequency of individual ADRs when undergoing AD treatment.

Variable	Mean	SD	Minimum	Maximum
Dizziness	-2.01	0.97	-4.00	0.00
Nausea	-1.99	1.03	-4.00	0.00
Sleep disorders	-1.93	1.09	-4.00	0.00
Palpitations	-1.83	1.00	-4.00	0.00
Headache	-1.76	1.00	-4.00	0.00
Reduced libido	-1.24	1.04	-3.00	1.00
Fainting	-1.24	0.89	-2.00	2.00
Seizures	-1.09	0.90	-2.00	2.00
Visual disturbances	-0.81	1.00	-3.00	1.00
Hallucinations	-0.81	1.12	-2.00	2.00
Hair loss	-0.76	1.04	-2.00	2.00
Fever	-0.43	0.85	-3.00	0.00
Mania	-0.22	1.13	-2.00	2.00
Loss of appetite	-0.15	1.09	-2.00	2.00
Weight gain	0.55	1.10	-2.00	2.00

M = mean, SD = standard deviation.

The estimated frequency of the ADR loss of appetite was not significantly different from the corresponding package-insert frequency category (*t*(196) = -1.89, *p* = 0.061, *d* = -0.14). The participants estimated the occurrence of the ADR weight gain to be significantly higher than stated in the package insert (*t*(200) = 7.03, *p* = < 0.001, *d* = 0.50). For 13 ADRs, participants selected package-insert frequency categories that were significantly lower than the corresponding reference categories (Table 5).

**Table 5:** Comparison of the estimated and actual frequencies of ADRs.

Variable	Test value	M	SD	t(df)	*p*	95% CI		d
						LB	UB	
Palpitations	5	3.18	1.00	-25.74 (201)	< 0.001	-1.96	-1.68	-1.81
Sleep disorders	5	3.07	1.09	-24.81 (195)	< 0.001	-2.08	-1.78	-1.77
Dizziness	5	2.99	0.97	-29.54 (201)	< 0.001	-2.15	-1.88	-2.08
Nausea	5	3.01	1.03	-27.97 (207)	< 0.001	-2.13	-1.85	-1.94
Headache	5	3.24	1.00	-24.65 (197)	< 0.001	-1.90	-1.62	-1.75
Fever	4	1.75	0.85	-37.02 (193)	< 0.001	-2.37	-2.13	-2.66
Visual disturbances	4	2.24	1.00	-24.62 (195)	< 0.001	-1.90	-1.61	-1.76
Reduced libido	4	3.57	1.04	-5.75 (193)	< 0.001	-0.57	-0.28	-0.41
Hallucinations	3	2.19	1.12	-10.35 (202)	< 0.001	-0.97	-0.66	-0.73
Fainting	3	1.74	0.89	-20.37 (203)	< 0.001	-1.39	-1.14	-1.43
Seizures	3	1.91	0.90	-17.01 (197)	< 0.001	-1.22	-0.96	-1.21
Mania	3	2.78	1.13	-2.81 (200)	0.005	-0.38	-0.07	-0.20
Loss of appetite	3	2.85	1.09	-1.89 (196)	0.061	-0.30	0.01	-0.14
Weight gain	3	3.55	1.10	7.03 (200)	< 0.001	0.39	0.70	0.50
Hair loss	3	2.24	1.04	-10.21 (193)	< 0.001	-0.91	-0.62	-0.73

M = mean, SD = standard deviation, CI = Confidence Interval. LB = Lower Bound. UB = Upper Bound. d = Cohen’s d.

### MAB

**Fig. 2:**
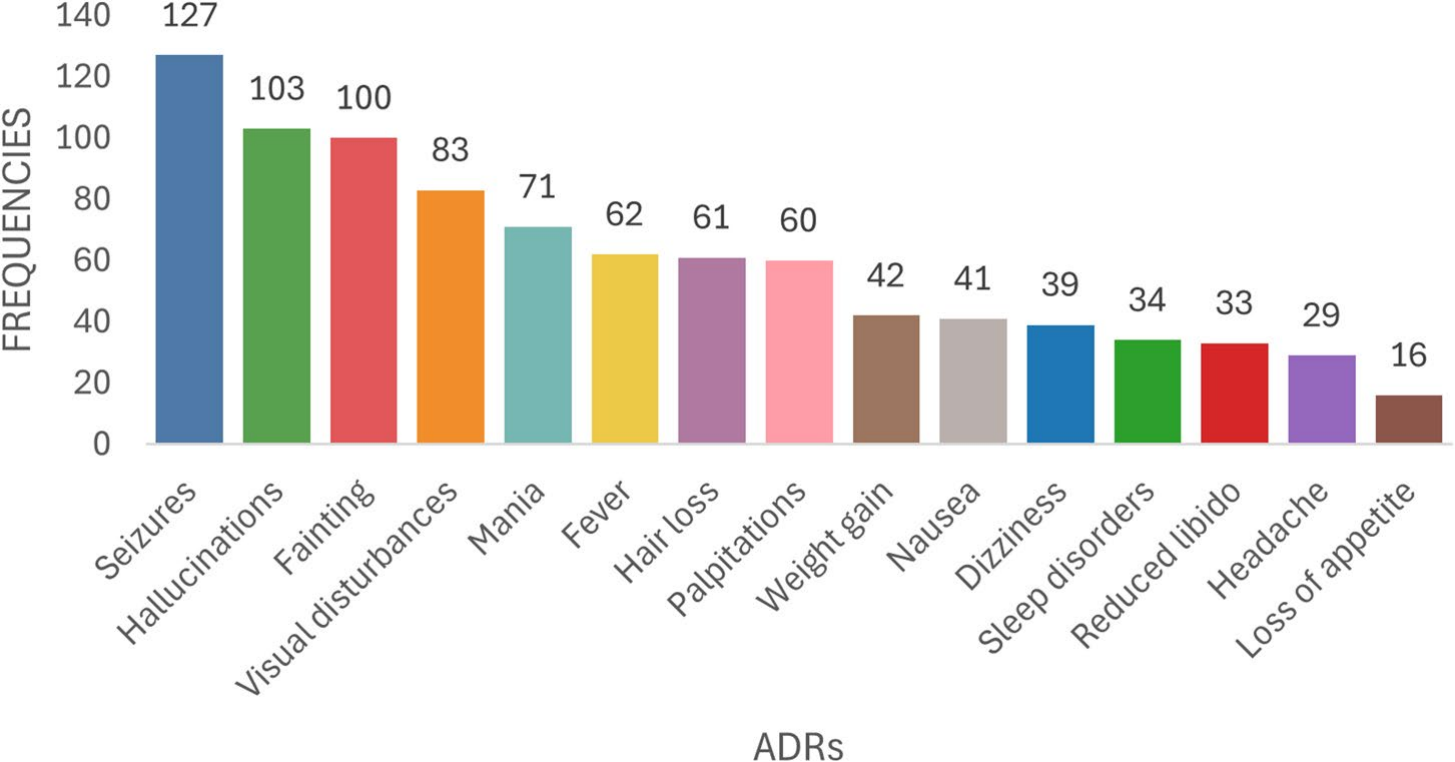
Frequency of the response option, “I would not accept this ADR under any circumstances” for each ADR.

Participants could indicate that they would not accept an ADR under any circumstances (Fig. 2). For example, *n* = 127 participants stated that they would, under no circumstances, accept the ADR seizures.

Among the remaining participants who were willing to accept ADRs, the MAB differed for each one. For example, participants would accept the ADR loss of appetite if their depressive symptoms improved by *M* = 14.88 (*SD* = 9.23) on the MADRS, which corresponded to the lowest MAB (Table 6). For the ADR seizures, participants reported the highest MAB (*M* = 23.51, *SD* = 8.82).

**Table 6:** MAB of each ADR.

Variable	*N*	M	SD	Minimum	Maximum
Seizures	69	23.51	8.82	0.00	34.00
Hallucinations	62	23.47	9.35	1.00	34.00
Fainting	59	23.22	9.38	0.00	34.00
Hair loss	104	21.08	9.31	0.00	34.00
Visual disturbances	90	20.88	9.58	0.00	34.00
Mania	92	19.64	9.81	0.00	34.00
Sleep disorders	135	19.43	8.93	0.00	34.00
Fever	114	19.39	8.97	2.00	34.00
Nausea	124	19.03	8.59	0.00	34.00
Weight gain	123	18.86	9.44	0.00	34.00
Palpitations	118	18.77	8.94	2.00	34.00
Dizziness	125	18.37	8.92	0.00	34.00
Headache	131	18.31	8.63	0.00	34.00
Reduced libido	153	17.88	9.35	0.00	34.00
Loss of appetite	134	14.88	9.23	0.00	34.00

N = number of participants, M = mean, SD = standard deviation.

Exclusion of negative values.

### Group comparisons

#### Effectiveness of Ads

Participants with a history of clinical depression believed ADs to be more effective than those with no such histories (112.30 vs. 92.31, *U* = 4181.00, *Z* = -2.422, *p* < 0.05). Sex, education level, and use of ADs had no influence on this estimation (**Supplementary Table 5**).

#### ADRs

Compared to males, females estimated some ADRs to be more frequent (palpitations, dizziness, headache, visual disturbances, reduced libido, fainting, weight gain, and hair loss) (**Supplementary Table 6**). Previous history of depression led those participants to estimate the ADR sleep disorders as more frequent (106.61 vs. 90.55, *U* = 4014.50, *Z* = -2.055, *p* < 0.05). There were no significant differences regarding education level. Participants who had never taken ADs rated the occurrence of some ADRs (fever, hallucination, seizures, dizziness and mania) as more frequent than those who had experiences with AD treatment (**Supplementary Table 6**).

#### MAB

Females required a higher MAB than males to accept the ADRs of weight gain (68.80 vs. 47.90, *U* = 1096.00, *Z* = -3.051, *p* < 0.05) and seizures (39.79 vs. 28.77, *U* = 398.00, *Z* = -2.272, *p* < 0.05). Participants with histories of clinical depression demanded a higher MAB to accept the ADRs of nausea (69.94 vs. 55.06, *U* = 1460.50, *Z* = -2.311, *p* < 0.05) and dizziness (71.25 vs. 53.47, *U* = 1390.00, *Z* = − 0.742, *p* < 0.05). Participants with a lower education level required a higher MAB for the ADR of hair loss (77.40 vs. 49.85, *U* = 221.00, *Z* = -2.751, *p* < 0.05). There were no statistically significant differences in the other MABs with respect to sex, education level, history of clinical depression, or experience with ADs (**Supplementary Table 7**).

## Discussion

Our study showed that the participants exhibit a pronounced tendency to overestimate the effectiveness of ADs. The combination therapy treatment approach was rated as the most effective and believed that an untreated depressive episode would worsen. For 13 of the 15 ADRs investigated, participants selected significantly lower frequency categories than the corresponding reference categories in the package inserts, whereas for the ADR weight gain, they selected a significantly higher category. More than half of participants indicated they would not accept the ADR seizure. Only prior history of clinical depression, but not experience with AD treatment, influenced its perceived effectiveness and MAB.

On average, participants reported that depressive symptoms would have to improve by 18 points on the MADRS for them to be willing to take an AD and accept the potential occurrence of ADRs. This finding is consistent with Hengartner and Plöderl’s^13^ suggestion that the benefit required by patients is higher when potential ADRs are considered (MAB) than when estimating the MID without ADRs^13^.

This finding corroborates the legitimacy of the debate concerning the clinical relevance of ADs, especially in the context of ensuring transparent, shared decision-making processes based on probabilities and effect sizes of benefits and harms as the foundation of evidence-based medicine. A comparison of the estimated effectiveness with the actual effectiveness of ADs shows a clear discrepancy. If the required MID is substantially higher than the actual effectiveness of ADs, this raises questions regarding the high volume of psychopharmaceutical prescriptions^10^ and how benefits and risks of ADs are communicated and understood in clinical practice.

Previous studies often exclusively included participants without prior experience of the treatment investigated; consequently, differences based on treatment experience were not examined^21,35^. By contrast, our study included participants with and without AD experience. Even so, no differences in the perceived frequency of ADRs were observed. So, it seems that lived treatment experience alone may not substantially improve knowledge of ADR frequency. However, our findings do not allow conclusions to be drawn about the quality, content, or adequacy of physician–patient communication or the information provided during prescribing consultations.

Various studies have shown that advertising can influence not only patients^36^, but also physicians. Contact with pharmaceutical representatives is known to increase the volume of prescriptions, regardless of proper indication^37,38^. A potential explanation for the overestimation of the ADR of weight gain could be inaccurate media coverage on the relationship between ADs and weight gain. Reports of weight gain caused by ADs exist not only in research literature^39–41^, but also in popular health magazines such as *Apotheken Umschau* or *Deutsche Apotheker Zeitung*^42,43^. As demonstrated in a Danish study, the media can influence public opinion on ADs by its selection of topics, its emphasis on specific aspects, and the manner of reporting^44^. It is plausible that in the current study, the ADR of weight gain was thus more familiar to participants than other ADRs, which triggered a recognition bias and a tendency to overestimate the risk of this ADR.

Our results suggests that the MAB may be worth discussing and incorporating into treatment decisions.

During consultation, patients’ expectations should be discussed and potentially relativized so that they can be more fully involved in the decision-making process. The MAB could also be considered as a potential component of future decision-support approaches.

Based on our findings, we encourage physicians and patients alike, to learn about and discuss benefits and risks of AD treatment, as well as to consider treatment options in general, to avoid overtreatment. Currently, it appears that not only patients, but also physicians tend to overestimate the benefits, and underestimate the potential harm of (AD) treatment^19,20^. We plan on replicating this study with a cohort of general practitioners to compare the perspectives.

### Strengths and limitations

To our knowledge, the current study is the first to explore the perspective of members of the public about the potential harms and benefits of AD treatment. The inclusion of patients with AD experience in our study is a strength, as these were often neglected in previous studies. Although Citalopram was selected as an example, the test value for effectiveness was based on a review with 28 RCTs and thus to a wider range of ADs^25^ so that the results can also be transferred to other ADs. The benchmark of 2.99 MADRS points is based on mostly short-term observation in randomized controlled trials. This should be distinguished from the present survey scenario, which assessed perceived effectiveness over a six-month period. Longer-term outcomes may be more relevant to patients which motivated our choice of time frame.

Our broad recruitment strategy resulted in an unknown number of recipients (response rate cannot be calculated) and the non-representative nature of the sample, which predominantly consists of females aged 25 to 54 years with a high level of education. However, since more women present with clinical depression than men, this puts the high proportion of women in our sample into relative perspective. Approximately half of the participants had previously experienced or were currently experiencing a depressive episode, which notably, is higher than the estimated prevalence and lifetime prevalence of depressive disorders in Germany^2,3^. Despite the non-representative nature of our sample, our findings suggest that participants tended to overestimate the effectiveness of ADs and to assign lower frequency categories to many ADRs than those reported in package inserts. As these reference values are based on broad ordinal categories as presented in package inserts rather than directly observed incidence rates, and do not represent precise risk estimates, the findings should be interpreted with caution. They reflect differences in perceived frequency classifications rather than perceptions of absolute risk. Nevertheless, the overall pattern is consistent with previous research showing that treatment benefits are often perceived more favorably than potential harms^19^. A limitation of this study is that we did not assess how participants interpreted the likelihood of the presented ADRs. Participants may have differed in whether they perceived the ADRs as hypothetical, probable, or certain outcomes. Such differences in interpretation could have influenced the MAB thresholds reported in this study.

Our analysis is based on self-reported information, which might be affected by recall bias. We therefore cannot rule out (self-)selection bias due to particularly high motivation to participate among personally-affected people. Furthermore, we cannot rule out that participants took part in the survey more than once, since in order to ensure anonymity, we did not use cookies or check IP addresses. Given the exploratory nature of this study, we did not adjust for multiple comparisons. Our findings should be interpreted as exploratory and hypothesis-generating.

## Conclusion

The findings suggest that patient expectations, as well as the balance between benefits and risks of ADs, may warrant greater attention in clinical consultations. Future research should investigate whether incorporating the concept of the MAB can support informed consent processes and enhance shared decision-making.

## Supplementary Information

Below is the link to the electronic supplementary material.

Supplementary Material 1

## Data Availability

The data generated and analysed during the current study are available in the osf repository (https://osf.io/m26pe).

## Abbreviations

ADs: Antidepressants
ADRs: Adverse drug reactions
MADRS: Montgomery Åsberg Depression Rating Scale
MID: Minimal important difference
MAB: Minimum acceptable benefit
