# Supplementary material for: Public thresholds for antidepressant effectiveness and adverse drug reaction rates: a cross-sectional study
Source: Sci Rep. 2026 Jul 15;16:22283. doi: 10.1038/s41598-026-62116-y (PMC13373223; doi:10.1038/s41598-026-62116-y)
Supplement: Supplementary file 1 — Supplementary Material 1 [file 41598_2026_62116_MOESM1_ESM.docx]

**Supplementary Table 1.** Checklist for Reporting Results of Internet E-Surveys (CHERRIES)

| ***Checklist Item*** | ***Explanation*** | ***Page Number*** |
| --- | --- | --- |
| Describe survey design | Describe target population, sample frame. Is the sample a convenience sample? (In “open” surveys this is most likely.) | 5 |
| IRB approval | Mention whether the study has been approved by an IRB. | 5 |
| Informed consent | Describe the informed consent process. Where were the participants told the length of time of the survey, which data were stored and where and for how long, who the investigator was, and the purpose of the study? | 5 |
| Data protection | If any personal information was collected or stored, describe what mechanisms were used to protect unauthorized access. | 5 |
| Development and testing | State how the survey was developed, including whether the usability and technical functionality of the electronic questionnaire had been tested before fielding the questionnaire. | 5 |
| Open survey versus closed survey | An “open survey” is a survey open for each visitor of a site, while a closed survey is only open to a sample which the investigator knows (password-protected survey). | 5 |
| Contact mode | Indicate whether or not the initial contact with the potential participants was made on the Internet. (Investigators may also send out questionnaires by mail and allow for Web-based data entry.) | 5 |
| Advertising the survey | How/where was the survey announced or advertised? Some examples are offline media (newspapers), or online (mailing lists – If yes, which ones?) or banner ads (Where were these banner ads posted and what did they look like?). It is important to know the wording of the announcement as it will heavily influence who chooses to participate. Ideally the survey announcement should be published as an appendix. | 5 |
| Web/E-mail | State the type of e-survey (eg, one posted on a Web site, or one sent out through e-mail). If it is an e-mail survey, were the responses entered manually into a database, or was there an automatic method for capturing responses? | 5 |
| Context | Describe the Web site (for mailing list/newsgroup) in which the survey was posted. What is the Web site about, who is visiting it, what are visitors normally looking for? Discuss to what degree the content of the Web site could pre-select the sample or influence the results. For example, a survey about vaccination on a anti-immunization Web site will have different results from a Web survey conducted on a government Web site | 5 |
| Mandatory/voluntary | Was it a mandatory survey to be filled in by every visitor who wanted to enter the Web site, or was it a voluntary survey? | 5 |
| Incentives | Were any incentives offered (eg, monetary, prizes, or non-monetary incentives such as an offer to provide the survey results)? | 5 |
| Time/Date | In what timeframe were the data collected? | 5 |
| Randomization of items or questionnaires | To prevent biases items can be randomized or alternated. | 7, 8 |
| Adaptive questioning | Use adaptive questioning (certain items, or only conditionally displayed based on responses to other items) to reduce number and complexity of the questions. | 5, 6 |
| Number of Items | What was the number of questionnaire items per page? The number of items is an important factor for the completion rate. | 5 |
| Number of screens (pages) | Over how many pages was the questionnaire distributed? The number of items is an important factor for the completion rate. | 5 |
| Completeness check | It is technically possible to do consistency or completeness checks before the questionnaire is submitted. Was this done, and if “yes”, how (usually JAVAScript)? An alternative is to check for completeness after the questionnaire has been submitted (and highlight mandatory items). If this has been done, it should be reported. All items should provide a non-response option such as “not applicable” or “rather not say”, and selection of one response option should be enforced. | NA |
| Review step | State whether respondents were able to review and change their answers (eg, through a Back button or a Review step which displays a summary of the responses and asks the respondents if they are correct). | 5 |
| Unique site visitor | If you provide view rates or participation rates, you need to define how you determined a unique visitor. There are different techniques available, based on IP addresses or cookies or both. | 18 |
| View rate (Ratio of unique survey visitors/unique site visitors) | Requires counting unique visitors to the first page of the survey, divided by the number of unique site visitors (not page views!). It is not unusual to have view rates of less than 0.1 % if the survey is voluntary. | 18 |
| Participation rate (Ratio of unique visitors who agreed to participate/unique first survey page visitors) | Count the unique number of people who filled in the first survey page (or agreed to participate, for example by checking a checkbox), divided by visitors who visit the first page of the survey (or the informed consents page, if present). This can also be called “recruitment” rate. | 18 |
| Completion rate (Ratio of users who finished the survey/users who agreed to participate) | The number of people submitting the last questionnaire page, divided by the number of people who agreed to participate (or submitted the first survey page). This is only relevant if there is a separate “informed consent” page or if the survey goes over several pages. This is a measure for attrition. Note that “completion” can involve leaving questionnaire items blank. This is not a measure for how completely questionnaires were filled in. (If you need a measure for this, use the word “completeness rate”.) | 9 |
| Cookies used | Indicate whether cookies were used to assign a unique user identifier to each client computer. If so, mention the page on which the cookie was set and read, and how long the cookie was valid. Were duplicate entries avoided by preventing users access to the survey twice; or were duplicate database entries having the same user ID eliminated before analysis? In the latter case, which entries were kept for analysis (eg, the first entry or the most recent)? | 18 |
| IP check | Indicate whether the IP address of the client computer was used to identify potential duplicate entries from the same user. If so, mention the period of time for which no two entries from the same IP address were allowed (eg, 24 hours). Were duplicate entries avoided by preventing users with the same IP address access to the survey twice; or were duplicate database entries having the same IP address within a given period of time eliminated before analysis? If the latter, which entries were kept for analysis (eg, the first entry or the most recent)? | 18 |
| Log file analysis | Indicate whether other techniques to analyze the log file for identification of multiple entries were used. If so, please describe. | NA |
| Registration | In “closed” (non-open) surveys, users need to login first and it is easier to prevent duplicate entries from the same user. Describe how this was done. For example, was the survey never displayed a second time once the user had filled it in, or was the username stored together with the survey results and later eliminated? If the latter, which entries were kept for analysis (eg, the first entry or the most recent)? | NA |
| Handling of incomplete questionnaires | Were only completed questionnaires analyzed? Were questionnaires which terminated early (where, for example, users did not go through all questionnaire pages) also analyzed? | 9 |
| Questionnaires submitted with an atypical timestamp | Some investigators may measure the time people needed to fill in a questionnaire and exclude questionnaires that were submitted too soon. Specify the timeframe that was used as a cut-off point, and describe how this point was determined. | NA |
| Statistical correction | Indicate whether any methods such as weighting of items or propensity scores have been used to adjust for the non-representative sample; if so, please describe the methods. | 8 |

This checklist has been modified from Eysenbach G. Improving the quality of Web surveys: the Checklist for Reporting Results of Internet E-Surveys (CHERRIES). J Med Internet Res. 2004 Sep 29;6(3):e34 [erratum in J Med Internet Res. 2012; 14(1): e8.]. Article available at [https://www.jmir.org/2004/3/e34](https://www.jmir.org/2004/3/e34/)/; erratum available <https://www.jmir.org/2012/1/e8/>. Copyright ©Gunther Eysenbach. Originally published in the [Journal of Medical Internet](http://www.jmir.org) Research, 29.9.2004 and 04.01.2012.

**Supplementary Table 2.** Survey

| STUDY DESCRIPTION | | |
| --- | --- | --- |
| 1.1. | **Online-Survey: Treatment of mental illnesses from the perspective of citizens**  Thank you for taking the time to participate in our survey.  **What is it about?** We would like to assess how laypersons evaluate the benefits and potential risks of antidepressants in the treatment of depression.  **Who is conducting this study?** The Institute of General Medicine at the University Hospital Erlangen.  **Who can participate?** People aged 18 and over  **How does the study work?** Please set aside 10-15 minutes to participate in the following survey.  If you have any questions, please send an email to [***contact]***  If you would like to participate in the study, please click on Next Page. | |
| 1.2. | **Data protection**  Your data will be collected anonymously, i.e. you will not be asked for your name at any point. Anonymisation will take place immediately, and it will not be possible to assign or trace the data afterwards. The results of the study will be published in anonymised form. The fully anonymised data will be made available to the public via the internet database Open Science Framework (osf.io) after the end of the project.  **Voluntariness**  Participation in the survey is voluntary. You can decide to stop taking part in this study at any time without giving a reason, without any consequences for you.  The data collected and personal communications made in the course of this study will be treated confidentially.  Detailed information about the study and the detailed privacy policy can be downloaded here: [***link to study information and privacy policy]*** | |
| 1.4 | I hereby confirm that I am of legal age. | - Yes - No, I am not of legal age |
| 1.5 | I hereby agree to the privacy policy. | - Yes - No, I want to stop taking part in the survey [*end*] |

| 2. SOZIODEMOGRAPHIC DATA | | |
| --- | --- | --- |
|  | Description/Item | Response options |
| 2.1 | Please state your age: | - 18-24 years - 25-39 years - 40-54 years - 55-69 years - 70+ years |
| 2.2 | Please state your gender: | - female - male - diverse |
| 2.3 | Please select the highest level of education you have attained to date. | - without certificate - secondary certificate - intermediate certificate - completed apprenticeship - (specialised) A-levels - (specialised) university degree - no information provided |

| 3. ILLNESS-RELATED DATA | | |
| --- | --- | --- |
|  | Description/Item | Response options |
| 3.1 | Are you currently suffering from depression? | - Yes - No |
| 3.2 | By whom was this diagnosed? | - By my general practitioner - By my psychotherapist - By my psychiatrist - By another specialist - My depression has not been officially diagnosed |
| 3.3 | Have you been told what severity level you have (mild, moderate or severe depression)? | - Yes - No |
| 3.4 | Which severity level do you have? | - Mild depression - Moderate depression - Severe depression |
| 3.5 | Are you undergoing treatment for depression? | - Yes - No |
| 3.6 | What type of treatment is it? | - Psychotherapy - Pharmacotherapy (taking an antidepressant) - Combination therapy (psychotherapy and pharmacotherapy) - Other [text field] |
| 3.7 | Has your doctor informed you about the effectiveness of antidepressants? | - Yes - No |
| 3.8 | Have you ever suffered from depression? | - Yes - No |
| 3.9 | Have you undergone treatment for depression back then? | - Yes - No |
| 3.10 | What type of treatment was it? | - Psychotherapy - Pharmacotherapy (taking an antidepressant) - Combination therapy (psychotherapy and pharmacotherapy) - Other [text field] |
| 3.11 | Have you been informed about the effectiveness of antidepressants? | - Yes - No |

| 4. EFFECTIVENESS | | |  |
| --- | --- | --- | --- |
| 4.0 | *Please immerse yourself in the following scenario:*  *You have been suffering from a reduced drive for 6 weeks. Activities that you normally enjoy no longer give you any pleasure. You also have difficulty concentrating on things. You have problems falling asleep in the evening. You also wake up several times during the night. You are further troubled by unfounded self-reproaches. You have lost your appetite, so you have already lost 3 kilograms in the last few weeks.*  *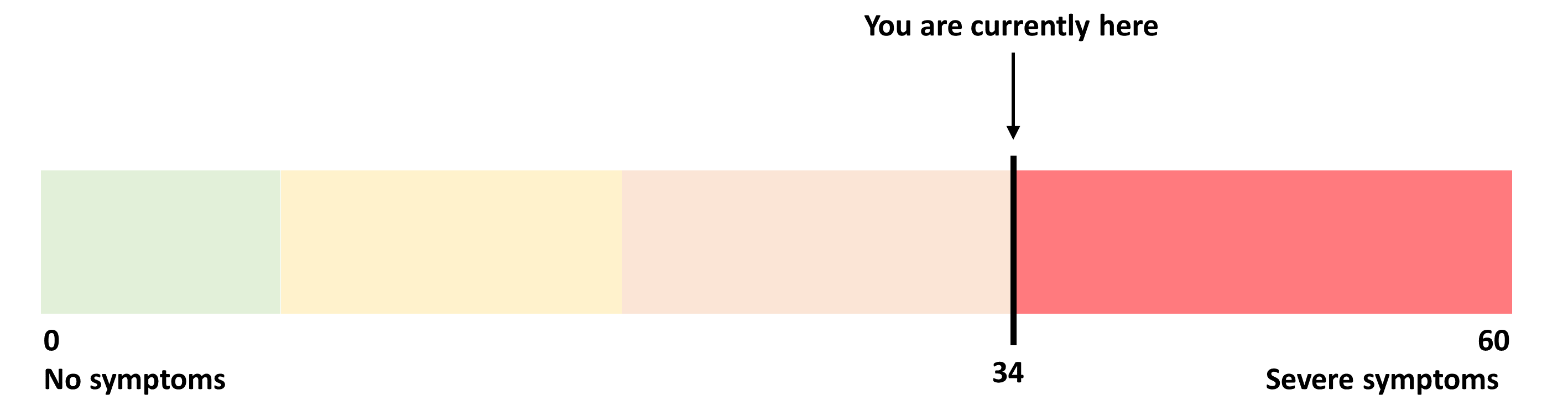*  Based on your symptoms, you currently have a score of 34 on the depression scale, which is on the threshold of severe symptoms. The scale ranges from 0 = no symptoms to 60 = severe symptoms.  There are various treatment options. For the scenario described, please estimate where you would be on the scale from 0 = no symptoms to 60 = severe symptoms after 6 months of treatment. Please set the respective  score using the slider.  To move the slider, please click on it first. This is your subjective assessment, so there is no right or wrong answer. | | |
|  | Description/Item | Response options | |
| 4.1 | Where are you on the scale after taking an antidepressant for 6 months? | Scale with a slide bar from 0-60, with the slider set at 34. | |
| 4.2 | Where are you on the scale after 6 months of psychotherapy? | As in 4.1 | |
| 4.3 | Where are you on the scale after 6 months of combination therapy (taking an antidepressant in combination with psychotherapy)? | As in 4.1 | |
| 4.4 | Where are you on the scale after 6 months without any treatment? | As in 4.1 | |

| 5. SIDE EFFECTS (risk assessment) | | |  |
| --- | --- | --- | --- |
| 5.0 | How likely do you think it is that the following side effects will occur when taking an antidepressant?  Please estimate the likelihood of the occurrence of each listed side effect. There is no right or wrong answer. | |  |
|  | Description/Item | Response options | |
| 5.1 | Heart palpitations, trembling, increased sweating | - very rare - rare - occasionally - frequent - very frequent | |
| 5.2 | Sleep disorders | As in 5.1 | |
| 5.3 | Dizziness | As in 5.1 | |
| 5.4 | Nausea, constipation | As in 5.1 | |
| 5.5 | Headaches | As in 5.1 | |
| 5.6 | Fever | As in 5.1 | |
| 5.7 | Visual disturbances | As in 5.1 | |
| 5.8 | Reduced libido | As in 5.1 | |
| 5.9 | Hallucinations | As in 5.1 | |
| 5.10 | Fainting | As in 5.1 | |
| 5.11 | Seizures | As in 5.1 | |
| 5.12 | Pathological euphoria (mania) | As in 5.1 | |
| 5.13 | Loss of appetite | As in 5.1 | |
| 5.14 | Weight gain | As in 5.1 | |
| 5.15 | Hair loss | As in 5.1 | |
|  | |  |  |

| 6. SIDE EFFECTS | | | |  |
| --- | --- | --- | --- | --- |
| 6.0 | Please think back to the scenario described above.  Based on your symptoms (reduced motivation, loss of pleasure, sleep difficulties, unfounded self-reproaches, loss of appetite), you score 34 points on the depression scale.  Your psychiatrist prescribes you an antidepressant, which you are to take daily.  How much would your symptoms have to improve for you to accept the respective side effect?  *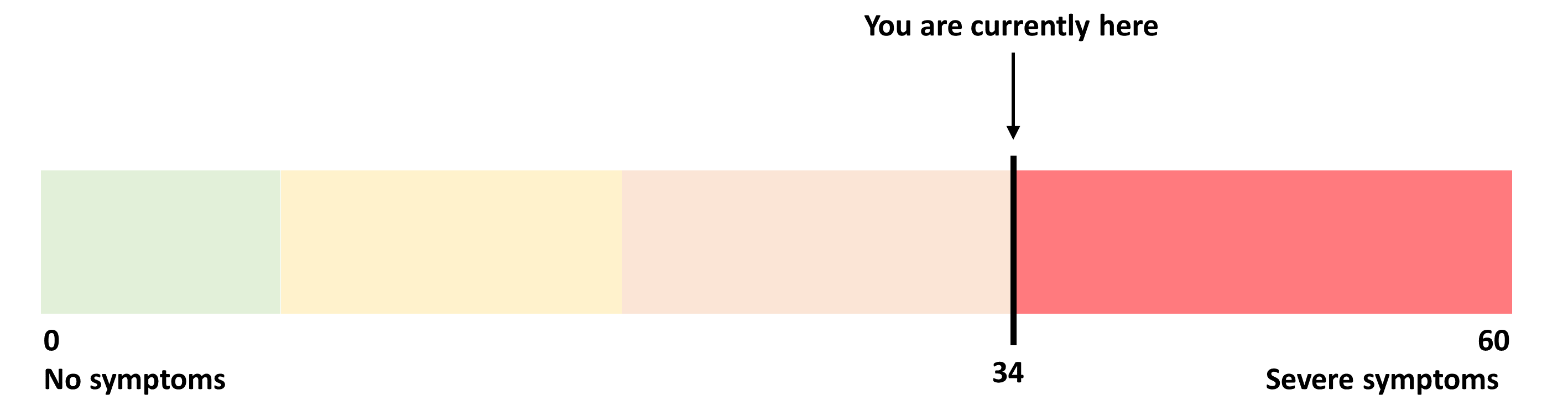*  Your starting point is 34 points. Please use the slider to set the new score that your symptoms would have to improve to. To move the slider, please click on it first.  If you would not accept a side effect under any circumstances, please check the corresponding box and do not adjust the slider. | | |  |
|  | Description/Item | | Response options | |
| 6.1 | Heart palpitations, trembling, increased sweating | | Improvement of symptoms to __ Points  Slide bar (from 0-60)  Scale labels:  0 = No symptoms  60 = Severe symptoms   - I would not accept this side effect under any circumstances. | |
| 6.2 | Sleep disorders | | As in 6.1 | |
| 6.3 | Dizziness | | As in 6.1 | |
| 6.4 | Nausea, constipation | | As in 6.1 | |
| 6.5 | Headaches | | As in 6.1 | |
| 6.6 | | Fever | As in 6.1 | |
| 6.7 | | Visual disturbances | As in 6.1 | |
| 6.8 | | Reduced libido | As in 6.1 | |
| 6.9 | | Hallucinations | As in 6.1 | |
| 6.10 | | Fainting | As in 6.1 | |
| 6.11 | | Seizures | As in 6.1 | |
| 6.12 | | Pathological euphoria (mania) | As in 6.1 | |
| 6.13 | | Loss of appetite | As in 6.1 | |
| 6.14 | | Weight gain | As in 6.1 | |
| 6.15 | | Hair loss | As in 6.1 | |

| 7. HEALTH LITERACY (HLS-EU-Q16) | | | |
| --- | --- | --- | --- |
| 7.0 | On a scale from very easy to very difficult, how easy do you think it is to... | |  |
|  | Description/Item | Response options | |
| 7.1 | ...find information about treatments for illnesses that affect you? | - Very easy - Easy - Difficult - Very difficult | |
| 7.2 | ... find out where to get professional help when you are ill? (physician, pharmacist, psychologist) | As in 7.1 | |
| 7.3 | ...understand what you physician is telling you? | As in 7.1 | |
| 7.4 | ...understand the instructions given by your doctor or pharmacist on how to take your prescribed medication? | As in 7.1 | |
| 7.5 | ...tell when you should get a second opinion from another physician? | As in 7.1 | |
| 7.6 | ...make decisions concerning your illness based on the information provided by your physician? | As in 7.1 | |
| 7.7 | ...follow the instructions given by your physician or pharmacist? | As in 7.1 | |
| 7.8 | ...find information about support options for mental health issues such as stress or depression? | As in 7.1 | |
| 7.9 | ...understand health warnings about behaviours such as smoking, lack of exercise or excessive drinking? | As in 7.1 | |
| 7.10 | ...understand why you need preventive medical check-ups? (early cancer detection, blood sugar test, blood pressure) | As in 7.1 | |
| 7.11 | …assess whether information about health risks in the media is reliable? (Television, internet or other media) | As in 7.1 | |
| 7.12 | ...decide how to protect yourself from illnesses based on information from the media? (newspapers, brochures, the internet or other media) | As in 7.1 | |
| 7.13 | ...find information about behaviours that are good for your mental well-being? (meditation, physical exercise, walking, pilates, etc.) | As in 7.1 | |
| 7.14 | ...understand health advice from family members or friends? | As in 7.1 | |
| 7.15 | ...understand information in the media about how you can improve your health? (internet, newspapers, magazines) | As in 7.1 | |
| 7.16 | ...assess which everyday habits are related to your health? (drinking and eating habits, exercise, etc.) | As in 7.1 | |

**Supplementary Table 3.** ADRs used in the present study

| **Very frequent** | **Frequent** | **Occasionally** |
| --- | --- | --- |
| - Palpitations, trembling, increased sweating - Sleep disorders - Dizziness - Nausea, constipation - Headache | - Fever - Visual disturbances - Reduced libido | - Hallucinations - Fainting - Seizures - Pathological euphoria - Loss of appetite - Weight gain - Hair loss |

**Supplementary Table 4.** Excluded number of cases for each ADR regarding the MABs

| **Variable** | ***N***  *MAB analysis* | ***n***  *Missings* | ***n***  *I would not accept this side effect under any circumstances* | ***n***  *Excluded* |
| --- | --- | --- | --- | --- |
| Seizures | 69 | 1 | 127 | 11 |
| Hallucinations | 62 | 35 | 103 | 8 |
| Fainting | 59 | 41 | 100 | 8 |
| Hair loss | 104 | 30 | 61 | 13 |
| Visual disturbances | 90 | 23 | 83 | 12 |
| Mania | 92 | 35 | 71 | 10 |
| Sleep disorders | 135 | 29 | 34 | 10 |
| Fever | 114 | 22 | 62 | 10 |
| Nausea | 124 | 28 | 41 | 15 |
| Weight gain | 123 | 29 | 42 | 14 |
| Palpitations | 118 | 20 | 60 | 10 |
| Dizziness | 125 | 35 | 39 | 9 |
| Headache | 131 | 34 | 29 | 14 |
| Reduced libido | 153 | 4 | 33 | 18 |
| Loss of appetite | 134 | 39 | 16 | 19 |

**Supplementary Table 5.** Group comparisons regarding the effectiveness of ADs

| **Grouping variable** | **Group** | **N** | **Mean rank** | **U** | **Z** | **P** |
| --- | --- | --- | --- | --- | --- | --- |
| Gender | Female | 143 | 106.17 | 3836 | -1.364 | .173 |
|  | Male | 61 | 93.89 |  |  |  |
| Experience with ADs | No | 170 | 103.36 | 2743 | -.469 | .639 |
|  | Yes | 34 | 98.18 |  |  |  |
| Experience with depression | No | 100 | 92.31 | 4181 | -2.422 | **.015** |
|  | Yes | 104 | 112.30 |  |  |  |
| Level of Education | Low | 22 | 106.11 | 1922.50 | -.304 | .761 |
|  | High | 182 | 102.06 |  |  |  |

| **Supplementary Table 6.** Group comparisons regarding the frequency of ADRs | | | | | | | |
| --- | --- | --- | --- | --- | --- | --- | --- |
| **ADR** | **Grouping variable** | **Group** | **N** | **Mean rank** | **U** | **Z** | **P** |
| Reduced libido | Gender | Female | 133 | 103.91 | 3204 | -2.459 | **.014** |
|  |  | Male | 61 | 83.52 |  |  |  |
|  | Experience with ADs | No | 163 | 95.56 | 2209.50 | -1.158 | .247 |
|  |  | Yes | 31 | 107.73 |  |  |  |
|  | Experience with depression | No | 96 | 93.92 | 4360 | -.921 | .357 |
|  |  | Yes | 98 | 101.01 |  |  |  |
|  | Level of Education | Low | 21 | 90.36 | 1666.50 | -.646 | .518 |
|  |  | High | 173 | 98.37 |  |  |  |
| Weight gain | Gender | Female | 138 | 107.63 | 3432.50 | -2.489 | **.013** |
|  |  | Male | 63 | 86.48 |  |  |  |
|  | Experience with ADs | No | 166 | 102.68 | 2625.50 | -.930 | .352 |
|  |  | Yes | 35 | 93.01 |  |  |  |
|  | Experience with depression | No | 96 | 97.02 | 4657.50 | -.967 | .334 |
|  |  | Yes | 105 | 104.64 |  |  |  |
|  | Level of Education | Low | 22 | 106.64 | 1845 | -.501 | .616 |
|  |  | High | 179 | 100.31 |  |  |  |
| Palpitations | Gender | Female | 139 | 109.51 | 3265.50 | -3.037 | **.002** |
|  |  | Male | 63 | 83.83 |  |  |  |
|  | Experience with ADs | No | 168 | 101.30 | 2823 | -.111 | .911 |
|  |  | Yes | 34 | 102.47 |  |  |  |
|  | Experience with depression | No | 102 | 99.57 | 4903.50 | -.497 | .619 |
|  |  | Yes | 100 | 103.47 |  |  |  |
|  | Level of Education | Low | 21 | 105.21 | 1822.50 | -.323 | .747 |
|  |  | High | 181 | 101.07 |  |  |  |
| Dizziness | Gender | Female | 139 | 108.03 | 3471 | -2.474 | **.013** |
|  |  | Male | 63 | 87.10 |  |  |  |
|  | Experience with ADs | No | 168 | 105.06 | 2257.50 | -2.020 | **.043** |
|  |  | Yes | 34 | 83.90 |  |  |  |
|  | Experience with depression | No | 99 | 104.02 | 4849.50 | -.629 | .529 |
|  |  | Yes | 103 | 99.08 |  |  |  |
|  | Level of Education | Low | 23 | 112.93 | 1795.50 | -1.046 | .296 |
|  |  | High | 179 | 100.03 |  |  |  |
| Headaches | Gender | Female | 137 | 108.43 | 2955 | -3.442 | **<.001** |
|  |  | Male | 61 | 79.44 |  |  |  |
|  | Experience with ADs | No | 165 | 99.61 | 2704.50 | -.063 | .950 |
|  |  | Yes | 33 | 98.95 |  |  |  |
|  | Experience with depression | No | 95 | 97.63 | 4714.50 | -.463 | .644 |
|  |  | Yes | 103 | 101.23 |  |  |  |
|  | Level of Education | Low | 21 | 112.64 | 1582.50 | -.1.164 | .244 |
|  |  | High | 177 | 97.94 |  |  |  |
| Fever | Gender | Female | 133 | 97.21 | 4018.50 | -.113 | .910 |
|  |  | Male | 61 | 98.12 |  |  |  |
|  | Experience with ADs | No | 161 | 101.68 | 1984 | -2.481 | **.013** |
|  |  | Yes | 33 | 77.12 |  |  |  |
|  | Experience with depression | No | 94 | 104.59 | 4034 | -1.847 | .065 |
|  |  | Yes | 100 | 90.84 |  |  |  |
|  | Level of Education | Low | 22 | 96.43 | 1868.50 | -.103 | .918 |
|  |  | High | 172 | 97.64 |  |  |  |
| Hallucinations | Gender | Female | 141 | 105.84 | 3829.50 | -1.463 | .143 |
|  |  | Male | 62 | 93.27 |  |  |  |
|  | Experience with ADs | No | 168 | 109.88 | 1617 | -4.359 | **<.001** |
|  |  | Yes | 35 | 64.20 |  |  |  |
|  | Experience with depression | No | 97 | 110.02 | 4363.50 | -1.937 | .053 |
|  |  | Yes | 106 | 94.67 |  |  |  |
|  | Level of Education | Low | 23 | 114.17 | 1790 | -1.099 | .272 |
|  |  | High | 180 | 100.44 |  |  |  |
| Fainting | Gender | Female | 142 | 107.71 | 3661.50 | -2.085 | **.037** |
|  |  | Male | 62 | 90.56 |  |  |  |
|  | Experience with ADs | No | 170 | 105.13 | 2443 | -1.554 | .120 |
|  |  | Yes | 34 | 89.35 |  |  |  |
|  | Experience with depression | No | 100 | 107.78 | 4672.50 | -1.367 | .172 |
|  |  | Yes | 104 | 97.43 |  |  |  |
|  | Level of Education | Low | 23 | 113.20 | 1835.50 | -1.007 | .314 |
|  |  | High | 181 | 101.14 |  |  |  |
| Mania | Gender | Female | 140 | 105.05 | 3703.50 | -1.543 | .123 |
|  |  | Male | 61 | 91.71 |  |  |  |
|  | Experience with ADs | No | 168 | 105.88 | 1953 | -2.769 | **.006** |
|  |  | Yes | 33 | 76.18 |  |  |  |
|  | Experience with depression | No | 101 | 108.66 | 4267.50 | -1.938 | .053 |
|  |  | Yes | 100 | 93.27 |  |  |  |
|  | Level of Education | Low | 22 | 97.43 | 1890.50 | -.315 | .753 |
|  |  | High | 179 | 101.44 |  |  |  |
| Loss of appetite | Gender | Female | 137 | 102.57 | 3620.50 | -1.376 | .169 |
|  |  | Male | 60 | 90.84 |  |  |  |
|  | Experience with ADs | No | 163 | 100.37 | 2547.50 | -.765 | .444 |
|  |  | Yes | 34 | 92.43 |  |  |  |
|  | Experience with depression | No | 98 | 100.07 | 4746 | -.272 | -.786 |
|  |  | Yes | 99 | 97.94 |  |  |  |
|  | Level of Education | Low | 23 | 107.11 | 1814.50 | -.751 | .452 |
|  |  | High | 174 | 97.93 |  |  |  |
| Hair loss | Gender | Female | 135 | 104.84 | 2991.50 | -2.872 | **.004** |
|  |  | Male | 59 | 80.70 |  |  |  |
|  | Experience with ADs | No | 161 | 99.38 | 2354.50 | -1.072 | .284 |
|  |  | Yes | 33 | 88.35 |  |  |  |
|  | Experience with depression | No | 96 | 96.41 | 4599.50 | -.279 | .781 |
|  |  | Yes | 98 | 98.57 |  |  |  |
|  | Level of Education | Low | 20 | 108.43 | 1521.50 | -.958 | .338 |
|  |  | High | 174 | 96.24 |  |  |  |
| Sleep disorders | Gender | Female | 134 | 103.36 | 3502.50 | -1.829 | .067 |
|  |  | Male | 62 | 87.99 |  |  |  |
|  | Experience with ADs | No | 165 | 100.48 | 2230.50 | -1.170 | .242 |
|  |  | Yes | 31 | 87.95 |  |  |  |
|  | Experience with depression | No | 99 | 90.55 | 4014.50 | -2.055 | **.040** |
|  |  | Yes | 97 | 106.61 |  |  |  |
|  | Level of Education | Low | 22 | 108.89 | 1685.50 | -.945 | .345 |
|  |  | High | 174 | 97.19 |  |  |  |
| Nausea | Gender | Female | 144 | 109.44 | 3897 | -1.850 | .064 |
|  |  | Male | 64 | 93.39 |  |  |  |
|  | Experience with ADs | No | 173 | 103.69 | 2887.50 | -.449 | .653 |
|  |  | Yes | 35 | 108.50 |  |  |  |
|  | Experience with depression | No | 102 | 104.96 | 5389 | -.113 | .910 |
|  |  | Yes | 106 | 104.06 |  |  |  |
|  | Level of Education | Low | 23 | 110.50 | 1989.50 | -.528 | .597 |
|  |  | High | 185 | 103.75 |  |  |  |
| Visual disturbances | Gender | Female | 136 | 104.61 | 3249.50 | -2.373 | **.018** |
|  |  | Male | 60 | 84.66 |  |  |  |
|  | Experience with ADs | No | 165 | 100.81 | 2176 | -1.377 | .169 |
|  |  | Yes | 31 | 86.19 |  |  |  |
|  | Experience with depression | No | 97 | 101.22 | 4538 | -.694 | .488 |
|  |  | Yes | 99 | 95.84 |  |  |  |
|  | Level of Education | Low | 23 | 117.17 | 1560 | -1.758 | .079 |
|  |  | High | 173 | 96.02 |  |  |  |
| Seizures | Gender | Female | 139 | 98.81 | 4004.50 | -.279 | .781 |
|  |  | Male | 59 | 101.13 |  |  |  |
|  | Experience with ADs | No | 164 | 102.91 | 2228 | -1.971 | **.049** |
|  |  | Yes | 34 | 83.03 |  |  |  |
|  | Experience with depression | No | 96 | 105.21 | 4348 | -1.455 | .146 |
|  |  | Yes | 102 | 94.13 |  |  |  |
|  | Level of Education | Low | 23 | 110.63 | 1756.50 | -1.060 | .289 |
|  |  | High | 175 | 98.04 |  |  |  |

| **Supplementary Table 7.** Group comparisons regarding the MABs | | | | | | | |
| --- | --- | --- | --- | --- | --- | --- | --- |
| **MAB** | **Grouping variable** | **Group** | **N** | **Mean rank** | **U** | **Z** | **P** |
| Reduced libido | Gender | Female | 108 | 76.91 | 2420.50 | -.038 | .970 |
|  |  | Male | 45 | 77.21 |  |  |  |
|  | Experience with ADs | No | 125 | 79.28 | 1465.50 | -1.346 | .178 |
|  |  | Yes | 28 | 66.84 |  |  |  |
|  | Experience with depression | No | 77 | 74.92 | 2766.00 | -.585 | .558 |
|  |  | Yes | 76 | 79.11 |  |  |  |
|  | Level of Education | Low | 10 | 71.00 | 655.00 | -.444 | .657 |
|  |  | High | 143 | 77.42 |  |  |  |
| Weight gain | Gender | Female | 83 | 68.80 | 1096.00 | -3.051 | **.002** |
|  |  | Male | 40 | 47.90 |  |  |  |
|  | Experience with ADs | No | 102 | 61.94 | 1064.50 | -.044 | .965 |
|  |  | Yes | 21 | 62.31 |  |  |  |
|  | Experience with depression | No | 63 | 57.47 | 1604.50 | -1.447 | .148 |
|  |  | Yes | 60 | 66.76 |  |  |  |
|  | Level of Education | Low | 9 | 72.56 | 418.00 | -.924 | .355 |
|  |  | High | 114 | 61.17 |  |  |  |
| Palpitations | Gender | Female | 81 | 61.74 | 1317.00 | -1.054 | .292 |
|  |  | Male | 37 | 54.59 |  |  |  |
|  | Experience with ADs | No | 94 | 61.23 | 965.50 | -1.088 | .277 |
|  |  | Yes | 24 | 52.73 |  |  |  |
|  | Experience with depression | No | 60 | 63.33 | 1510.50 | -1.237 | .216 |
|  |  | Yes | 58 | 55.54 |  |  |  |
|  | Level of Education | Low | 12 | 58.75 | 627.00 | -.080 | .936 |
|  |  | High | 106 | 59.58 |  |  |  |
| Dizziness | Gender | Female | 86 | 64.35 | 1561.00 | -.619 | .536 |
|  |  | Male | 39 | 60.03 |  |  |  |
|  | Experience with ADs | No | 105 | 64.22 | 922.00 | -.863 | .388 |
|  |  | Yes | 20 | 56.60 |  |  |  |
|  | Experience with depression | No | 58 | 53.47 | 1390.00 | -2.742 | **.006** |
|  |  | Yes | 67 | 71.25 |  |  |  |
|  | Level of Education | Low | 14 | 75.50 | 602.00 | -1.372 | .170 |
|  |  | High | 111 | 61.42 |  |  |  |
| Headaches | Gender | Female | 98 | 66.97 | 1522.00 | -.505 | .614 |
|  |  | Male | 33 | 63.12 |  |  |  |
|  | Experience with ADs | No | 108 | 65.80 | 1220.00 | -.133 | .894 |
|  |  | Yes | 23 | 66.96 |  |  |  |
|  | Experience with depression | No | 63 | 63.64 | 1993.50 | -.685 | .493 |
|  |  | Yes | 68 | 68.18 |  |  |  |
|  | Level of Education | Low | 12 | 67.46 | 696.50 | -.140 | .889 |
|  |  | High | 119 | 65.85 |  |  |  |
| Fever | Gender | Female | 79 | 57.72 | 1365.00 | -.108 | .914 |
|  |  | Male | 35 | 57.00 |  |  |  |
|  | Experience with ADs | No | 97 | 56.79 | 755.50 | -.550 | .582 |
|  |  | Yes | 17 | 61.56 |  |  |  |
|  | Experience with depression | No | 59 | 52.24 | 1312.00 | -1.764 | .078 |
|  |  | Yes | 55 | 63.15 |  |  |  |
|  | Level of Education | Low | 15 | 66.50 | 607.50 | -1.133 | .257 |
|  |  | High | 99 | 56.14 |  |  |  |
| Hallucinations | Gender | Female | 37 | 32.73 | 417.00 | -.655 | .512 |
|  |  | Male | 25 | 29.68 |  |  |  |
|  | Experience with ADs | No | 54 | 32.73 | 149.50 | -1.401 | .161 |
|  |  | Yes | 8 | 23.19 |  |  |  |
|  | Experience with depression | No | 31 | 27.68 | 362.00 | -1.674 | .094 |
|  |  | Yes | 31 | 35.32 |  |  |  |
|  | Level of Education | Low | 10 | 35.35 | 221.50 | -.739 | .460 |
|  |  | High | 52 | 30.76 |  |  |  |
| Fainting | Gender | Female | 43 | 29.34 | 315.50 | -.489 | .625 |
|  |  | Male | 16 | 31.78 |  |  |  |
|  | Experience with ADs | No | 50 | 30.14 | 218.00 | -.149 | .882 |
|  |  | Yes | 9 | 29.22 |  |  |  |
|  | Experience with depression | No | 29 | 28.95 | 404.50 | -.465 | .642 |
|  |  | Yes | 30 | 31.02 |  |  |  |
|  | Level of Education | Low | 10 | 38.70 | 158.00 | -1.769 | .077 |
|  |  | High | 49 | 28.22 |  |  |  |
| Mania | Gender | Female | 63 | 48.32 | 799.00 | -.965 | .334 |
|  |  | Male | 29 | 42.55 |  |  |  |
|  | Experience with ADs | No | 75 | 46.41 | 631.00 | -.066 | .948 |
|  |  | Yes | 17 | 46.88 |  |  |  |
|  | Experience with depression | No | 45 | 42.22 | 865.00 | -1.508 | .132 |
|  |  | Yes | 47 | 50.60 |  |  |  |
|  | Level of Education | Low | 12 | 48.54 | 455.50 | -.285 | .776 |
|  |  | High | 80 | 46.19 |  |  |  |
| Loss of appetite | Gender | Female | 93 | 65.70 | 1739.00 | -.813 | .416 |
|  |  | Male | 41 | 71.59 |  |  |  |
|  | Experience with ADs | No | 116 | 66.71 | 952.50 | -.600 | .548 |
|  |  | Yes | 18 | 72.58 |  |  |  |
|  | Experience with depression | No | 68 | 68.92 | 2147.50 | -.432 | .666 |
|  |  | Yes | 66 | 66.04 |  |  |  |
|  | Level of Education | Low | 13 | 75.27 | 685.50 | -.763 | .445 |
|  |  | High | 121 | 66.67 |  |  |  |
| Hair loss | Gender | Female | 68 | 56.13 | 977.00 | -1.691 | .091 |
|  |  | Male | 36 | 45.64 |  |  |  |
|  | Experience with ADs | No | 88 | 52.71 | 685.50 | -.167 | .867 |
|  |  | Yes | 16 | 51.34 |  |  |  |
|  | Experience with depression | No | 53 | 48.33 | 1130.50 | -1.440 | .150 |
|  |  | Yes | 51 | 56.83 |  |  |  |
|  | Level of Education | Low | 10 | 77.40 | 221.00 | -2.751 | **.006** |
|  |  | High | 94 | 49.85 |  |  |  |
| Sleep disorders | Gender | Female | 93 | 69.59 | 1805.50 | -.703 | .482 |
|  |  | Male | 42 | 64.49 |  |  |  |
|  | Experience with ADs | No | 114 | 70.60 | 901.00 | -1.801 | .072 |
|  |  | Yes | 21 | 53.90 |  |  |  |
|  | Experience with depression | No | 69 | 67.33 | 2230.50 | -.205 | .837 |
|  |  | Yes | 66 | 68.70 |  |  |  |
|  | Level of Education | Low | 11 | 65.73 | 657.00 | -.202 | .840 |
|  |  | High | 124 | 68.20 |  |  |  |
| Nausea | Gender | Female | 83 | 65.31 | 1468.50 | -1.240 | .215 |
|  |  | Male | 41 | 56.82 |  |  |  |
|  | Experience with ADs | No | 104 | 62.62 | 1028.00 | -.082 | .935 |
|  |  | Yes | 20 | 61.90 |  |  |  |
|  | Experience with depression | No | 62 | 55.06 | 1460.50 | -2.311 | **.021** |
|  |  | Yes | 62 | 69.94 |  |  |  |
|  | Level of Education | Low | 10 | 82.90 | 366.00 | -1.875 | .061 |
|  |  | High | 114 | 60.71 |  |  |  |
| Visual disturbances | Gender | Female | 60 | 46.53 | 838.00 | -.532 | .595 |
|  |  | Male | 30 | 43.43 |  |  |  |
|  | Experience with ADs | No | 75 | 46.57 | 482.00 | -.874 | .382 |
|  |  | Yes | 15 | 40.13 |  |  |  |
|  | Experience with depression | No | 45 | 42.86 | 893.50 | -.962 | .336 |
|  |  | Yes | 45 | 48.14 |  |  |  |
|  | Level of Education | Low | 12 | 51.96 | 390.50 | -.922 | .357 |
|  |  | High | 78 | 44.51 |  |  |  |
| Seizures | Gender | Female | 39 | 39.79 | 398.00 | -2.272 | **.023** |
|  |  | Male | 30 | 28.77 |  |  |  |
|  | Experience with ADs | No | 60 | 35.79 | 222.50 | -.850 | .396 |
|  |  | Yes | 9 | 29.72 |  |  |  |
|  | Experience with depression | No | 35 | 31.74 | 481.00 | -1.374 | .170 |
|  |  | Yes | 34 | 38.35 |  |  |  |
|  | Level of Education | Low | 12 | 43.58 | 239.00 | -1.637 | .102 |
|  |  | High | 57 | 33.19 |  |  |  |
